# Diagnostic Dilemma in a Young Woman with Acute Headache: Delayed Diagnosis of Third Ventricular Colloid Cyst with Hydrocephalus

**DOI:** 10.1155/2015/180404

**Published:** 2015-08-09

**Authors:** Jasem Y. Al-Hashel, Azza A. H. Rady, Doaa Y. Soliman, Periasamy Vembu

**Affiliations:** ^1^Department of Neurology, Ibn Sina Hospital, P.O. Box 25427, 13115 Safat, Kuwait; ^2^Department of Medicine, Faculty of Medicine, Kuwait University, P.O. Box 24923, 13110 Safat, Kuwait; ^3^Department of Neurology, Cairo University, Egypt

## Abstract

*Objectives*. To highlight the importance of early diagnosis of colloid cyst of the third ventricle and its early management. *Clinical Presentation and Intervention*. This is a young lady who presented with sudden onset headache. She attended a local clinic and also her area hospital. Her diagnosis was delayed several hours due to a diagnostic dilemma initially. No surgical intervention was tried since the patient developed early signs of brainstem coning by the time she was seen by neurosurgeon. Patient died after few days in spite of intensive ICU measures. *Conclusion*. Sudden onset headache in young adults should be looked at carefully. Early imaging is mandatory to prevent mortality.

## 1. Introduction

Colloid cysts of the third ventricle are benign cysts but sometimes can be life-threatening. Headache is the most common symptom in 75% of patients. Headache can be constant, intermittent, or migrainous in nature [1]. The definite cause of this lethal phenomenon is still a matter of debate. Acute blockage of the CSF fluid may cause hydrocephalus that leads to rostrocaudal herniation of the brainstem and causes brain death [2]. Here we report a young woman with severe headache and recurrent vomiting since early morning. She was diagnosed with colloid cyst with hydrocephalus by imaging (CT/MRI) at the time when her general condition deteriorated and finally resulted in her death unfortunately.

## 2. Clinical Presentation and Intervention

22-year-old healthy lady, 5 days prior to her admission, developed global, bitemporal headache that is associated with nausea and vomiting. She was seen in a local clinic and then in her area hospital. She was treated with simple analgesic drugs but the headache did not subside but in fact it was increasing in intensity. Five days, she went to another main area hospital because the headache was intolerable.

She was treated with analgesics, antiemetic, and sedative injections. Patient became drowsy but still arousal. An immediate neurological consultation was requested. In the past 5 days, there was no brain imaging requested.

**Figure 1:**
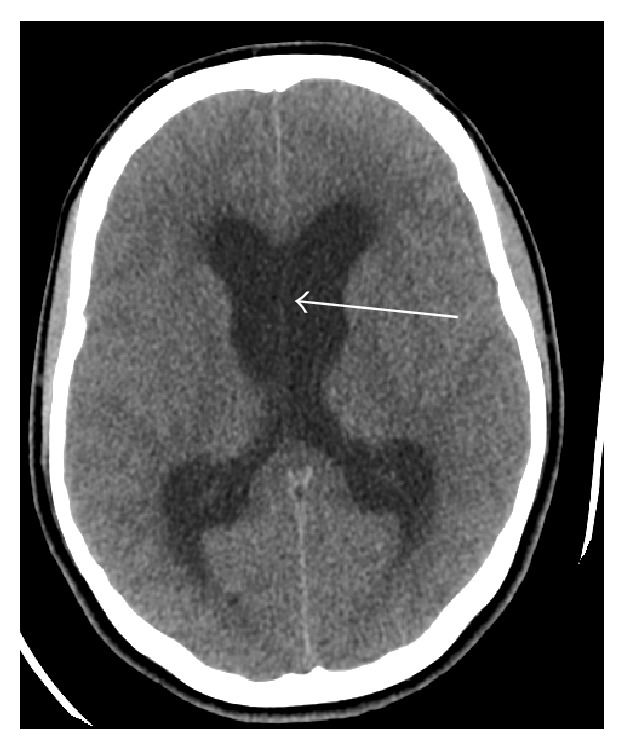
Plain axial CT scan of brain-showing severe obstructive lateral ventricle hydrocephalus shown by white arrow with signs of brain edema.

At the time she was assessed by a neurologist, she was very drowsy but conscious, moving her limbs, holding her head, and shouting due to pain. Her pupils were 3 mm reacting to light with spontaneous full eye movements. Fundi were normal also. Deep tendon jerks were very brisk all over and she had extensor planters. Patient was admitted and urgent CT brain was requested. The CT brain showed severe obstructive hydrocephalus; see Figure 1. Neurosurgeon inserted external ventricular drainage (EVD) immediately. The patient was transferred to the ICU for further treatment. Few hours later, her condition deteriorated more with spontaneous eye opening, and pupils were 4 mm but not reacting to light with only minimal response to painful stimuli.

**Figure 2:**
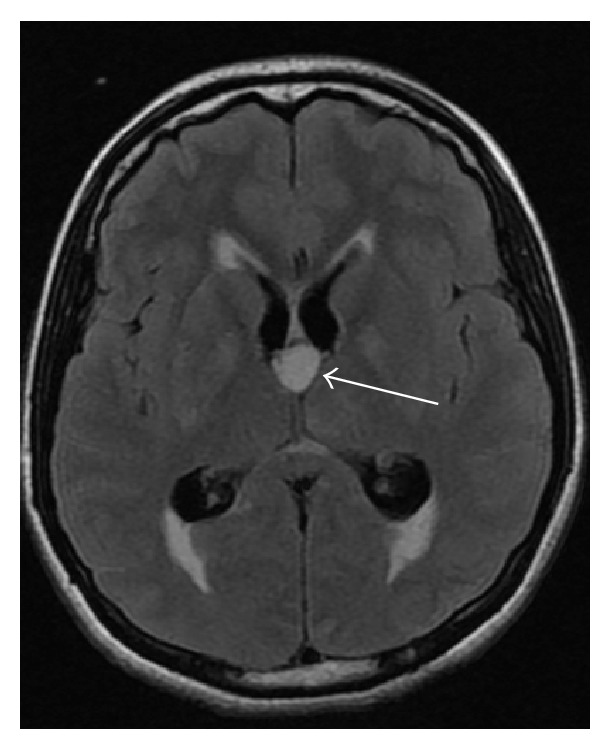
MRI brain FLAIR image axial view showing round shaped mass in the third ventricle, with enlarged both lateral ventricles shown by white arrows.

**Figure 3:**
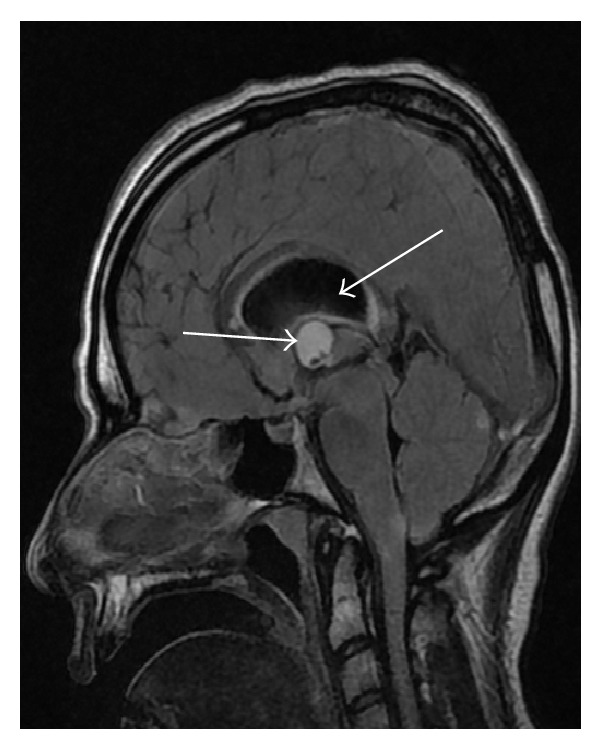
MRI brain of the same patient, sagittal view showing oval mass in the third ventricle at foramen of Monro, with hydrocephalus shown by arrows.

Two hours later, an urgent MRI brain with contrast was arranged. There was a well-defined nonenhanced oval lesion at the foramen of Monro, with high signal intensity at T2 and FLAIR study. The lesion is about 12.2 × 14.7 × 12.3 mm in size with obstructive hydrocephalus. The neurosurgeon deferred any surgical intervention since patient's general condition was poor at that time; see Figures 2 and 3.

On the next day, the patient's GCS was 3/10 and she was unconscious. A follow-up CT brain revealed brain edema with functioning EVD tube. She was started on antiedema measures with dexamethasone, mannitol, hypertonic saline, and hyperventilation. On the third day after admission, without sedation, the patient showed minimal response to pain over the limbs, with pupils 4 mm not reacting to light.

A follow-up CT brain showed increasing brain edema. On day 4, the blood pressure of the patient dropped and she was maintained on inotropic support.

**Figure 4:**
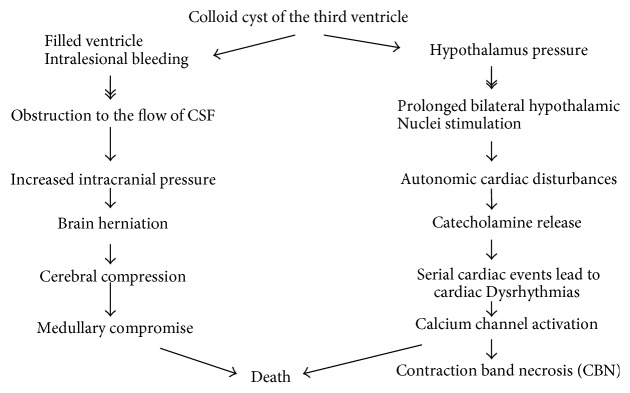
Mechanism of sudden death of patients with colloid cyst: hypothalamic structures which are involved in cardiovascular control are located close to the walls of the third ventricle which is most frequent anatomical site of the colloid cyst. It is adopted by Turillazzi et al. [4].

On day 5, patient developed dilated pupils, 7 mm in size, not reactive to light. A follow-up CT brain showed more brain edema with loss of white and grey mater differentiation; see Figure 4.

On day 7, caloric and apnea tests were done after stopping sedation. Pupils were 7 mm not reacting to light with absent vestibuloocular and corneal reflexes. Patient was declared to be brain-dead unfortunately 7 days after her admission.

## 3. Discussion

Colloid cyst was first described by Wallman in 1858 and then by Dandy in 1922. They detected these cysts with ventriculography and pneumoventriculography [3]. Colloid cysts of the third ventricle are benign cysts, but sometimes they can cause sudden death in young adults. Colloid cysts of the third ventricle are rare. They account for 0.5–2.0% of all intracranial tumors and 10–20% of all intraventricular tumors [4].

Colloid cyst is the most common third ventricular mass in adults. Many colloid cysts are asymptomatic and are diagnosed incidentally during routine imaging usually for headache. Headache is the most common symptom, seen in 75% of patients. Headache may be constant, intermittent, or migrainous in nature. The headache is due to transient obstruction secondary to a ball valve mechanism at the foramen of Monro [4]. The mechanism(s) of death in colloid cyst is still a controversial subject. The acute deterioration is possibly initiated by increase in sagittal sinus pressure, which initiates acute brain swelling and ultimately a series of events leading to death [4]. The headache is more in the early morning or shortly after patient wakes up and is intensified by changes in the position of the head or the body. The other symptoms are vertigo, drop attacks, and sudden attack of leg weakness. Sudden death is the most extreme presentation of the disease. The incidence of sudden death caused by brain tumor ranges from 0.16% to 3.2% and the majority of the cases are due to colloid cyst of the third ventricle [2, 4].

The definite cause of this lethal phenomenon is still a matter of debate. Acute blockage of the CSF fluid may cause hydrocephalus, which leads to rostrocaudal herniation of the brainstem causing brain death [5]. Sudden death was reported to occur in about 10% of patients with colloid cyst of the third ventricle. Symptomatic colloid cyst of the 3rd ventricle had a risk of acute deterioration in 34% and a mortality rate of 12% [6]. Many cysts are clinically silent; symptoms may result from persistent or intermittent obstruction of foramina of Monro [2, 5]. This can lead to acute lateral ventricle dilatation (hydrocephalus) with manifestations of intracranial hypertension which can lead to death. Death may also be secondary to reflex cardiac effects that is mediated through the compression of the hypothalamus by the cyst. Humphries et al. reported 98 cases of sudden death due to colloid cyst of the third ventricle [6].

A circadian variation in human CSF production has been demonstrated using MRI phase imaging.

A minimum production of volume (21.7 mL/hr) at 18.00 p.m. with a peak production is twice the day production at 02.00 a.m. (42.2 mL/hr) [4]. Most of the cases died in the early morning due to this peak of production. In most of the reported cases of death, since no images were done, the mechanisms of death were multiple. Instant herniation due to increased intracranial pressure from the hydrocephalus and the reflex cardiac effects mediated through the hypothalamus are most plausible [4].

Also, cardiac arrest due to hypothalamic stimulation by the colloid cyst may result in death.

Sudden increase in the intracranial pressure in the third ventricle may cause neurogenic cardiac stunning secondary to hyperacute and intense neurogenic sympathetic activation [5]. The cyst can fill the ventricle or obstruct the flow of CSF resulting in prominent hydrocephalus. Acute ventricular hydrocephalus with intracranial hypertension and brain herniation can result in cerebral compression, medullary compression (respiratory compromise), and eventually death. Sudden death, also, may be related to acute cyst swelling which causes acute obstructive hydrocephalus and intracranial hypertension. The acute swelling may result from intralesional hemorrhage [6, 7].

Hypothalamic structures, which are involved in nonendocrine and autonomic regulation, play a key role in controlling cardiac function. They are located close to the wall of the third ventricle. This may suggest that reflex cardiac effects due to compression of the hypothalamic cardiovascular regulatory centers by the cyst may explain the sudden death in patients with colloid cysts [8, 9]. It is well known that stimulation of the hypothalamus can lead to autonomic cardiovascular disturbances. Bilateral prolonged stimulation of the hypothalamus produces cardiac alterations similar to that produced by catecholamine injection which is called contraction band necrosis (CBN) [4].

## 4. Conclusion

This case reminds us about the importance of early brain imaging in children and young adults when they present with headache and vomiting, especially if this is the first presentation and there is no previous history of migraine. If a CT brain was done early in this patient, it may facilitated the diagnosis and hopefully prevent the mortality of this young lady. This patient had typical history of severe headache and vomiting starting in the early morning; the diagnosis was delayed due to late imaging and also delayed referral to neurologist for taking their opinion. Colloid cyst of the third ventricle should be in our differential diagnosis in a patient with acute onset of headache especially when other etiologies have been ruled out. In patients with third ventricle colloid cyst, even if it is asymptomatic or small cyst, surgical removal of the colloid cysts of the third ventricle is mandatory [10].
